# Mental health outcome measures in the Australian context: what is the problem represented to be?

**DOI:** 10.1186/s12888-022-04459-0

**Published:** 2023-01-10

**Authors:** Candice Oster, Suzanne Dawson, Jocelyn Kernot, Sharon Lawn

**Affiliations:** 1 Present Address: Caring Futures Institute, College of Nursing & Health Sciences, Flinders University, GPO Box 2100, Bedford Park, Adelaide, 5001 South Australia; 2 School of Allied health and Practice, University of Adelaide, Adelaide, 5005 South Australia; 3 Central Adelaide Local Health Network, Adelaide, South Australia; 4 Allied Health & Human Performance, University of South Australia, GPO Box 2471, Adelaide, 5001 South Australia; 5 Lived Experience Australia Ltd, PO Box 12, Oaklands Park, 5046 South Australia; 6 College of Medicine and Public Health, Flinders University, GPO Box 2100, Bedford Park, Adelaide, 5001 South Australia

**Keywords:** Routine outcome measures, Mental health policy, Policy analysis

## Abstract

**Background:**

There is growing interest in the use of routine outcome measures (ROM) in mental health services worldwide. Australia has been at the forefront of introducing ROM in public mental health services, with the aim of improving services and consumer outcomes.

**Methods:**

An in-depth policy and document analysis was conducted using Carol Bacchi’s ‘What is the problem represented to be?’ approach to critically analyse the use of ROM. This approach was used to identify and analyse the problem representations relating to the need for, and the choice of, outcome measures in Australian public mental health services, and the potential consequences of policy and practice. Data included in the analysis were seven policy documents, four reports on the introduction of outcome measures in Australia, the Australian Mental Health Outcomes and Classifications Network website, and the content of the outcome measures themselves.

**Results:**

Two dominant representations of the ‘problem’ were identified: 1) the ‘problem’ of mental health service quality and accountability, relating to the need for mental health outcome measures; and 2) the ‘problem’ of addressing deficits in biopsychosocial functioning of mental health consumers, which relates to the choice of outcome measures. Framing the ‘problem’ of mental health outcomes in these ways locates the problem within individual health providers, services, and consumers, ignoring the broader socioeconomic conditions underpinning mental health and effective service provision.

**Conclusions:**

This critical analysis of the introduction and use of ROM in public mental health services in Australia highlights the need to consider the role of the social determinants of mental health, mental health service funding, and recovery-oriented care in ensuring services are meeting consumer needs and expectations. Broader governmental engagement is central to genuine change and opportunities.

## Introduction

The introduction of routine outcome measures (ROM) to evaluate mental health services emerged in the late 1990s [1]. The purpose of ROM is to determine if treatment is having the desired benefits for the consumer and to assist with ongoing service planning and management [1–3]. ROMs are intended to act as a key driver of reform, underpinned by the notion that “You can’t improve what you can’t measure” ([4], p180). Outcome measures typically involve consumers or clinicians rating mental health symptoms and general distress; everyday functioning; perceived recovery; quality of life; and service satisfaction [1–3]. In current mental health settings clinicians may be required to integrate ROM and consumer feedback into daily practice, with outcome monitoring also appearing prominently in mental health policy [1–3].

Australia was one of the first countries to introduce routine outcome measurement of mental health services. This was “linked with concerns that services are accountable, are engaged in a constant endeavour to improve the quality of their work, and use the available resources in the best and fairest way possible” ([5], p. i). The process began with Australia’s first national Mental Health Policy in 1992 [6] and has continued through Australia’s subsequent policy and Mental Health Plans [7–12], with the use of ROMs made mandatory at a national level in 2000 [3, 13]. Since then, the introduction of ROM in public mental health services has been driven by States and Territories who are responsible for the delivery of public sector mental health services. The Australian Mental Health Outcomes and Classifications Network (AMHOCN) was formed by the Australian government in 2003 with the aim of supporting States and Territories in improving the collection and use of routine outcome measurement by services [13, 14]. Public sector mental health services, which include a range of inpatient, ambulatory, and community services, are funded by State and Territory governments and available free of charge. The Australian Mental Health Outcomes and Classifications Network provides guidance to organisations to embed ROM in these services [13].

The ROM implemented by States and Territories are collectively titled the National Outcomes and Casemix Collection (NOCC), which include a mixture of clinician and consumer rated tools (varying for different age groups) [13, 14]. The ROM tools were selected according to evidence-based literature, stakeholder consultation (consumer and clinician), and service trials [13], and also developed specifically for the purpose of outcome data collection under Australian policy in the case of the Health of the Nation Outcome Scale [15]. For the adult population, the national clinician rated outcomes are the Health of the Nation Outcome Scale (HoNOS, measuring mental health and social functioning) and the Life Skills Profile 16 (LSP-16, measuring psychosocial disability). Consumer rated outcomes vary across States and Territories and may include the Mental Health Inventory 38 (MHI, measuring psychological distress and wellbeing), Behavior & Symptom Identification Scale 32 (BASIS-32, measuring mental health symptoms), and the Kessler − 10+ (K-10+, measuring non-specific psychological distress). Clinicians in public mental health settings are mandated to collect these outcomes at admission and discharge from the service and at regular intervals (approximately every 91 days), with data sent to the Australian Government for analysis and reporting by AMHOCN and the Australian Institute of Health and Welfare. Reports are made available to everyone (i.e., general public, clinicians, etc.) on the AMHOCN websites report portal, with a ‘decision support tool’ developed for clinicians/services to allow comparison of their consumer outcomes with normative data of the population under care [13, 14].

While ROM is considered to be central to consumer-centred practice and in aiding decision making at the individual, service, and systems level [1], there have been a number of questions raised about their application internationally. For example, health care funders in the UK and USA are now placing increased emphasis on the use of ROM to support the distribution of funds [1, 16], raising questions about the appropriateness of using the same ROM across variable practice settings and diagnostic groups, with concerns that clinicians are being treated as commodities [1]. A further critique is the absence of diverse stakeholder consultation and shared decision making on the selection, implementation, and ongoing development of outcome tools. Without such consultation outcome measures may not be totally reflective of quality care, with the consumers’ goals/perspectives needing to be considered (e.g., a reduction of symptoms may not be the priority of all consumers) [1]. Furthermore, a systematic review by Gelkopf et al. [2] identified several barriers to the implementation of ROM in practice. These included: 1) clinicians questioning the value of these tools and the clinical benefits (due to implementation issues, impact on care, concerns about funding cuts and others’ perceptions of their skills, and lack of feedback of outcome analysis); 2) Issues around logistics and administration (e.g., staff turnover, time required to administer, lack of training around use, and lack of information technology (IT) support); and 3) low completion rates (attributed to points 1 & 2).

A Cochrane review conducted by Kendrick et al. [17] has furthermore found insufficient evidence that patient reported outcome measures improved treatment outcomes and ongoing management for people with common mental health disorders. This is reflected in the Australian context, where the extent to which outcome data has led to a culture of quality improvement and been used to improve mental health service quality has been questioned [18–21]. Lack of transparency in accountability for outcomes and lack of consistency in outcome measurement across programmes and services has also been identified [19], as well as the need to broaden reporting to incorporate social outcomes such as housing, education, employment, and social and family relationships [21, 22].

Given these concerns and identified barriers it is evident that further exploration of the implications and impact of ROM on mental health practice is required. This study utilises a novel approach to address this need by conducting an in-depth policy and document analysis, using Bacchi’s ‘What is the problem represented to be?’ analytic framework [23] to critically analyse the use of routine mental health outcome measures in the Australian context.

## Methods

Bacchi’s approach draws on the work of French philosopher Michel Foucault [24, 25], and is a form of critical analysis rather than a criticism of policy, analysing policy through a critical lens instead of attempting to explore whether the policy approach is correct [26]. As Foucault states:

A critique does not consist in saying that things aren’t good the way they are. It consists in seeing on what types of assumptions, of familiar notions, of established, unexamined ways of thinking the accepted practices are based. ([27], p. 456)

Bacchi’s approach therefore allows the exploration of the assumptions underpinning the introduction of outcome measures into mental health care and potential (intended and unintended) consequences. In doing so, the approach also opens up the possibility of thinking differently about outcome measurement in the mental health context.

Bacchi proposes that policies are problematising activities that “give shape to ‘problems’. They do not *address* them” ([23], p. x). Governments are therefore understood to produce problems rather than react to problems that exist independently in the world, with “particular representations of ‘problems’ [playing] a central role in how we are governed” ([23], p. x). This is not to suggest that there are not issues in society that need to be addressed. Instead, it highlights the importance of understanding how phenomena come to be understood as particular types of problems requiring specific policy solutions, and the implications of these problematisations.

### Data collection

Bacchi’s (2009) approach has been used to analyse a range of problem representations (e.g., disability in physical education teacher education [28]; sexual intimacy following intimate partner violence in the DSM-5 [29]; regulation of marriage migration to Norway [30]; parental substance abuse [31]), demonstrating the applicability of this approach beyond the analysis of policy documents to include other authoritative documents and reports [24, 29]. We therefore collected three types of data to explore the problem representations related to the introduction of ROM in mental health care in Australia. This included: 1) Australian mental health policy and plans in which outcome measures are referenced; and 2) documents and websites from those involved in the establishment of the current practices regarding the measurement of mental health outcomes in Australia, as well as 3) the measures themselves. The documents included in the analysis are presented in Table 1.

**Table 1:** Documents included in the analysis

Author (date)	Title
Australian Health Ministers (1992) [7]	First National Mental Health Plan
Australian Health Ministers (1992) [6]	National Mental Health Policy
Australian Health Ministers (1998) [8]	Second National Mental Health Plan
Australian Health Ministers (2003) [9]	Third National Mental Health Plan
Australian Health Ministers (2008) [10]	National Mental Health Policy
Australian Health Ministers (2009) [11]	Fourth National Mental Health Plan
Australian Health Ministers (2017) [12]	The Fifth National Mental Health and Suicide Prevention Plan
Australian Mental Health Outcomes Classification Network	https://www.amhocn.org
Australian Mental Health Outcomes Classification Network (2021) [14]	Mental Health National Outcomes and Casemix Collection: Overview of clinician-rated and consumer self-report measures
Andrews, Peters & Teesson (1994) [32]	The Measurement of Consumer Outcome in Mental Health
Stedman et al. (1997) [5]	Field Testing of Selected Measures of Consumer Outcome in Mental Health
Pirkis et al. (2005) [33]	Review of Standardised Measures Used in the National Outcomes and Casemix Collection (NOCC)
**Outcome measures**	Health of the Nation Outcome Scale (HoNOS); Life Skills Profile 16 (LSP-16); Mental Health Inventory 38 (MHI); Behavior & Symptom Identification Scale 32 (BASIS-32); Kessler − 10+ (K-10+); Children’s Global Assessment Scale (CGAS); Strengths and Difficulties Questionnaire (SDQ)

### Data analysis

Bacchi’s ‘What is the problem represented to be?’ approach involves asking six interrelated questions of the data collected for analysis, presented in Table 2.

**Table 2:** Bacchi’s six questions

1. What is the problem [of mental health outcome measurement] represented to be?	
2. What presuppositions or assumptions underpin this representation of the ‘problem’?	
3. How has this representation of the ‘problem’ come about?	
4. What is left unproblematic in this problem representation? Where are the silences? Can the ‘problem’ be thought about differently?	
5. What effects are produced by this representation of the ‘problem’?	
6. How/where has this representation of the ‘problem’ been produced, disseminated, and defended? How has it been (or could it be) questioned, disrupted, and replaced?	

The analysis involved familiarisation with the data by one of the authors (CO) followed by the development of initial responses to the six questions. The authors then met to discuss the early analysis and the question responses were refined based on the discussion. The process was repeated a further two times and the analysis finalised and agreed on.

### Terminology

There is considerable discussion and debate regarding how best to refer to those who receive health care [34], particularly mental health care [35]. Commonly used terms include ‘patient’, ‘client’, ‘consumer’, ‘service user’, ‘survivor’, and ‘people with lived experience’, each of which has its proponents and opponents [35]. Given the preference for the term ‘consumer’ in the Australian context, and in particular in groups representing those with lived experience of mental ill-health, the term ‘consumer’ is used herein.

## Results

Two dominant representations of the problem of mental health outcome measures were evident in the analysis. The first is the ‘problem’ of mental health service quality and accountability, which relates to the need for mental health outcome measures expressed in the documents. The second is the ‘problem’ of the requirement for mental health services to address deficits in functioning of mental health consumers, which relates to the choice of outcome measures. Each of these representations has varying histories and effects, presented below in relation to Bacchi’s six questions. We begin with a discussion of problem representations relating to the need for mental health measures, followed by a discussion of the choice of measures.

### Mental health service quality and accountability: the need for outcome measures

Analysis of the need for outcome measures in the documents in response to Bacchi’s six questions is summarised in Table 3 and discussed below.

**Table 3:** Analysis of the *need for* outcome measures reported in the documents

Bacchi’s Question	Response
1. What is the problem [of the need for mental health outcome measurement] represented to be?	• Lack of service quality
	• Lack of service value
	• Underperforming services and staff
2. What presuppositions or assumptions underpin this representation of the ‘problem’?	Managerial discourse:
	• Outcomes are attributable to mental health service intervention(s)
	• Lack of positive outcomes is due to low quality and performance of services and staff
	• Measuring outcomes will lead to improvement in service quality, efficiency, and accountability, which in turn will lead to improvement in consumer outcomes
	• Measuring and reporting outcomes, and comparing outcomes between services, will foster a culture of ongoing improvement within the services
	• Consumers and carers have access to a range of supports and services, the ability to choose the highest quality service, and will use ROM to select providers
	• Good mental health outcomes are defined by behaviours that can be assessed quantitatively
3. How has this representation of the ‘problem’ come about?	• Dominance of managerialism in healthcare
4. What is left unproblematic in this problem representation?	• Factors other than interventions implemented by mental health services that affect outcomes
	• The uncertainty of the relationship between measuring outcomes and quality improvement
5. What effects are produced by this representation of the ‘problem’?	Actual/Potential (unintended) consequences:
	Effects on health professionals:
	• Increased bureaucratic burden
	• Potential jobs lost
	• Being accountable to the measures rather than care provision
	• Loss of autonomy
	Effects on services:
	• Cost containment
	• Incentivised to ‘game the system’
	Effects on consumers/carers:
	• Reduction in quality of care
	• Undermining therapeutic relationship
	• Lack of services for vulnerable consumers
6a. How/where is this representation of the problem produced, disseminated, and defended?	Mental health policy and plans; AMHOCN website and documents; health service plans & models of care; academic literature
6b. How can it be questioned, disrupted, replaced?	Shift the focus to service funding, policy, and social norms to address broader determinants of mental health outcomes

### What is the problem [of the need for mental health outcome measurement] represented to be?

Answering Bacchi’s [23] first questions involves beginning with the solution that is presented in the documents and using the framing of the solution to make the problem explicit. There are often multiple problem representations that can be identified.

The need for clinical outcome measures (the solution) has been an integral part of Australia’s mental health policy and plans since 1992. The overarching framework in which the proposal of outcome measurement is presented is the need to “reduce functional impairment from mental disorder” ([7], p. 1) and improve outcomes for people with mental health problems and disorders. This is proposed to be achieved through ‘high quality’ mental health services and linking this to the measurement of outcomes as well as monitoring and accountability at the systematic level:

… services provided to people with mental health problems and mental illness should be monitored and evaluated to ensure that they are of high quality and achieving positive outcomes. ([10], p. 24)

This suggests the ‘problem’ to be one of the (potential) lack of high standards or quality of mental health care, situated within the broader concern about variability in service standards and quality discussed in mental health plans and policy since 1992 [36]. The first National Mental Health Plan, for example, noted that “the historical approach to mental health care has not always been conducive to achieving high standards” ([7], p. 29). Collecting and reporting on outcome measures was presented as a solution to improve quality of care, as discussed in the 2005 review of standardised measures used in the NOCC as follows:

The continued improvement of the quality and effectiveness of the treatment of people with a mental illness is one of the major objectives of the National Mental Health Strategy. The Strategy recognises that this objective can only be achieved through the development of sound information to support planning and service delivery. ([33], p. 2).

An additional problem representation relates to value for money and “cost-effectiveness” ([9], p. 15) on the part of government commissioning of services. The First National Mental Health Plan states under the section titled ‘Financial Accountability’:

... a comprehensive range of outcome measures, including efficiency and effectiveness measures, should be progressively developed; reporting on agreed outcome measures should commence in the 1993 National Report. ([7], np)

The problem in this statement is one of a lack of value in terms of wasted or misdirected (inefficient) use of resources in mental health services. A third problem representation relates to the concept of ‘performance indicators’, where outcomes measures are identified as an indicator of the performance of mental health services (and by implication the staff who work in these services). This implies the ‘problem’ of lack of performance.

Overall, then, outcome measures are proposed as the solution to the problem of mental health services that are lacking in quality and value and provided by services (and their staff) who are underperforming. The main mechanism by which outcome measures are proposed to solve the underlying problems is through making performance against the measures visible to staff, services, governments, consumers, and carers. This is proposed to allow comparison of performance across services and between services over time.

### What presuppositions or assumptions underpin this representation of the ‘problem’?

Answering Bacchi’s second question requires an analysis of the ‘conceptual logic’ that underpins the problem representations. By opening this up for examination, the purpose is not to make a determination of the veracity of presuppositions or assumptions. Instead, we aim to explore the “meanings that must be in place for a particular problem representation to ... make sense” ([23], p. 5). Identifying these meanings can be done through examination of binaries, key concepts, and categories.

The problem representation for the need for mental health outcome measures is underpinned by managerial discourse. Key concepts include ‘outcomes’, ‘quality’, ‘efficiency’, ‘accountability’, ‘benchmarking’, and ‘key performance indicators’, which in themselves create categories such as types of outcomes, degrees of quality and efficiency, and levels of performance. From this come the binaries of high versus low quality, positive versus negative outcomes, high versus low efficiency, good versus bad performance, and good versus bad outcomes.

Several assumptions underpin the presentation of the ‘problem’ of mental health care in managerial terms. A primary assumption is that outcomes are attributable to the interventions implemented by mental health services, which itself assumes that a lack of positive outcomes is due to low quality and performance on behalf of services and their staff [37]. A further assumption is that measuring outcomes will lead to an improvement in service quality, efficiency, and accountability and thereby improve consumer outcomes. The first report on the development of routine measures for Australian mental health services, for example, states:

This report … is focused on the evaluation of clinical practice and presumes that routine measurement will inform clinical practice, and directly and indirectly improve both the well-being of patients and the competence of clinicians. ([32], p. 12)

The Third Mental Health Plan ([9] p. 15) states:

Other approaches to increasing accountability at a service delivery level include ongoing implementation of consumer outcome measures that can be used routinely.

The plan also states that accountability “will be strengthened by the introduction of systems of public reporting by service organisations on key performance measures” (p. 61).

The use of accountability as a mechanism to improve service quality assumes that measuring and reporting outcomes, and comparing outcomes between services, will foster a culture of ongoing improvement within the services [38]. For example, the Fourth Mental Health Plan ([11], p. 52) states that developing key performance indicators and a benchmarking framework for comparing within and between services “is a key tool for promoting quality improvement” and will “build a culture of continuous quality improvement”. This is itself underpinned by an assumption that health providers and services “have the ability, and power, to implement changes” ([38], p. 243).

Reporting of outcome measures across services is furthermore promoted as a means of increasing consumer and carer choice regarding accessing quality mental health care. The Fifth Mental Health Plan ([12], p. 44) states that: “Information about the safety and quality of services will be available so that [consumers and carers] are able to make informed decisions about treatment, care and support.” This assumes that consumer and carers have access to a range of supports and services from which to choose and are in fact able to choose with regard to their treatment, care, and support [38]. It furthermore assumes that consumers and carers will use reported outcomes when selecting a provider [37].

The proposal of outcome measures in the documents furthermore privileges quantitative measures that allow comparison between services and over time as key performance indicators. This assumes good mental health outcomes are defined by “behaviors that can be easily assessed through quantitative techniques” ([38], p. 242).

### How has this representation of the ‘problem’ come about?

Bacchi’s third question explores the history of the problem representation and, in particular, how one representation of the ‘problem’ of quality mental health care came to be dominant. Prior to the introduction of the need for outcome measures in mental health, quality of care and positive outcomes for consumers prioritised medical expertise as the arbiter of quality care. The introduction of routine measurement and external surveillance of consumer outcomes challenges the dominance of medical expertise and shifts the focus from clinical judgement of outcomes to a managerial focus on quantitative outcomes for KPIs and benchmarking [39].

Managerialism is linked to the emergence and dominance of neoliberalism in Western countries, which is a partnership between government and market where market values are applied to the governance of areas such as healthcare, education, and welfare [40]. The emergence and growing proliferation of managerial discourse in health services occurred in the context of financial crises and increasing health demands in the 1980s and 1990s [41]. In Australian mental health care, this is evident in a report on field testing selected outcome measures, which cites “competing pressures for health care and social resources” ([5], p. i) as one of the reasons for the need to introduce the measures. This led to “a significant change in the boundaries of medical autonomy” ([42], p. 155) through a shift from the dominance of medical expertise and peer evaluation to increasing institutionalised regulation of service quality and efficiency.

Australia’s successive Mental Health Plans demonstrate managerialism in the evolution from the identification of the need for measuring outcomes [7], through the introduction of outcome measures into mental health services [8] and ongoing implementation [9], to the use of outcome measures for key performance indicators and benchmarking [11], and most recently, the proposal for increased transparency through enhanced public reporting [12]. This has been supported by initial government funding of $37 million to Australian states and territories to “support the development of clinical information systems that would further stimulate improvement in service quality, planning and policy development” ([18], p. 6). Further funding of $20 million was allocated with “the focus on improving capacity to apply the NOCC data to improve service quality” ([18], p. 6), followed by funding of AMHOCN on an ongoing basis to support the collection and use of routine outcome measurement by services.

### What is left unproblematic in this problem representation?

The purpose of the fourth question in Bacchi’s approach is to remind the reader that the ‘problem’ of quality mental health care can be conceptualised in different ways, and that these alternative perspectives are worthy of consideration. Two conceptualisations are excluded in the documents, namely the roles of factors other than interventions implemented by mental health services, such as consumer and carer actions and expertise, and social determinants of health, on consumer outcomes, and the uncertainty of the relationship between measuring outcomes and quality improvement.

As discussed above, a key assumption in the problem representation of the need for ROM is that outcomes are attributable to the interventions implemented by mental health services and staff. Yet this assumption has been questioned. For example, during early consultation on measuring outcomes in mental health, consumers and services providers “pointed out that where a measure did detect change in a person’s condition, the attribution of this change to any single factor, including intervention, may be problematic” ([5], p.2).

One factor that affects mental health outcomes outside of mental health settings is the actions of consumers themselves, as well as their carers and others in the broader community. The current problematisation views consumers and carers as affecting outcomes through the ability to compare the performance of services and choose those providing the highest quality of care. However, this might not be the case where there are limited services that are available and accessible [38]. Research has also found consumers and carers “rarely use performance data when selecting a provider” ([37], p. 57). Furthermore, by focusing on the role of clinicians and services, the actions and activities of consumers, peers, carers, and communities outside of mental health settings are marginalised in policy efforts to improve outcomes.

Social determinants of health are another factor affecting mental health outcomes that is left unproblematic [21, 43]. Social determinants of health are “the conditions in which people are born, grow, work, live, and age, as well as the set of forces and systems that shape daily life” ([44], p. 1). There is increasing recognition that mental health problems and outcomes of mental health care are strongly dependent on these conditions, systems, and forces [44, 45]. In the UK, for example, it is estimated that socio-economic conditions “could account for more than 40% of health performance in a given [health] authority” ([38], p. 244).

The assumption that measuring and reporting on outcomes will lead to improvement in quality has also been critiqued. As discussed earlier, a key concern in the evolution of the use of ROM in mental health in Australia has been a lack of “evidence that outcome measures routinely contribute to assessments of how to improve practice and clinical management at a service level” ([18], p. 17). Commentators have noted “Australia’s approach to accountability in mental health is overly focused on fulfilling governmental reporting requirements rather than using data to drive reform” ([21], p. 328). This suggests a missing piece in the relationship between measuring and reporting on outcomes and quality improvement. For example, financial constraints might limit the ability to improve services through actions such as increased expenditure on staff and services. Funding and commitment from all stakeholders are also needed to support efforts to build a quality improvement culture within services.

### What effects are produced by this representation of the ‘problem’?

Bacchi highlights that problematisations have multiple effects. These include discursive effects through limiting what can be thought or said in relation to the problem, subjectification effects through the constitution of particular subjects and subjectivities, and lived effects by their impact on people’s lives. Discursive effects can be seen in the discussion above about the ways in which the current problematisation excludes other ways of understanding the problem of quality mental health care and consequently less attention is paid to the role of factors such as broader systems and determinants of mental health.

In terms of subjectification effects, this is most clearly seen in the dichotomies discussed earlier and how these “set groups of people in opposition to each other” ([23], p. 16). Health professionals, for example, become effective or ineffective providers of mental health care within managerial discourse, with those deemed ineffective seen as responsible for the problem. This has lived effects on health professionals and their practice, such as an increased bureaucratic burden [16]. More seriously, a failure to achieve outcomes as defined by the measures might lead to “ineffectiveness [being] revealed or inappropriately deduced … and jobs lost” ([16], p. 315). The use of outcome measures as KPIs also has the potential to shift the focus of care from the client to the measure, such that health professionals are accountable to the measure rather than the care they provide. Health professionals have therefore expressed concern that ROM are “intrusive to clinical practice” ([2], p. 1) and “a threat to … clinical judgement, and therefore autonomy” ([46], p. 295). This is not to suggest that clinicians are infallible, nor that ineffectiveness should go unchecked, but rather to highlight the potential lived effects of the problem representation.

Mental health services are also affected by being divided into ‘good’ or ‘bad’ services in terms of the extent to which they meet KPIs related to outcome measures. A key concern for services is the potential for the measures to be used for “cost containment and service eligibility instead of service quality improvement” ([2], p. 1). Services are also affected by being incentivised to ‘game the system’ [37]. This was identified by consumers and service providers during early consultation, who noted the problematic attribution of change to the intervention (as discussed above) and expressed concern that “people may respond in a biased manner should resource of service delivery implications be attendant upon the results” ([5], p.2). Similarly, the Australian Productivity Commission’s Mental Health report ([20], p. 1221) identified concerns that “providers might ‘game’ the system, misreport or distort data to create a good impression, or focus attention on some performance measures at the expense of others” should benchmarking be introduced.

Effects on health professionals and services in turn have effects on the lives of consumers and their carers in relation to the extent to which the quality of care is improved through the introduction of routinely collected outcome measures. The relationship between consumer and health professional might also be affected, with consequent effects on care provision. In particular, a managerial approach to quality of care means that health professional might act “for the state or their employer … at the expense of working in partnership with clients” ([47], p. 248), leading to a transactional relationship between consumers and health professionals and services. Consumers have therefore expressed concern that the use of ROM functions as a bureaucratic exercise that can undermine the establishment of a therapeutic relationship with clinicians [48]. Thus, while Australia’s National Standards for Mental Health Service require a “commitment to improving the quality of care” ([22], p.3) in mental health services, potential unintended effects on clinical practice and ‘gaming the system’ might ultimately lead to a reduction in service quality rather than the quality improvement intended by policy.

Finally, there is also the risk that “services for the most vulnerable [could be] shut down” ([16], p. 315) should outcome measures be used to determine allocation of resources, particularly given the role of social determinants on mental health outcomes. This would exacerbate existing socioeconomic and geographic disparities in access to mental health services [49], a potential unintended consequence of the use of ROM.

### How/where is this representation of the ‘problem’ produced, disseminated and defended and how could it be questioned, disrupted and replaced?

Question six of Bacci’s analytic process explores how “particular problem representations reach their target audience and achieve legitimacy” ([23], p. 19). The representation of the problem of mental health outcomes as a lack of quality and value provided by services and their staff is produced, disseminated, and defended in a range of places. In addition to mental health policy and plans, it is also seen in reports relating to the introduction of ROM in mental health in Australia (e.g., [5, 32, 33]) and on the website of AMHOCN and the documents provided on the website (e.g., [14]). The representation is also produced and disseminated in Local Health Network (LHN) health service plans and models of care. There is some ‘questioning’ in these documents and websites. However, the focus is on which outcome measures to use rather than questioning the underlying assumptions of the problem representation.

Defence of the representation of the problem can furthermore be seen in academic literature. There are numerous studies, systematic reviews, and commentaries on the need for mental health outcome measures to improve the quality of mental health care. While the research does not provide definitive support for the effectiveness of this approach to improve quality, it generally does not challenge the need for outcome measures as a solution to the ‘problem’ of quality mental health care [1–3, 13, 17].

Bacchi’s sixth question also includes analysis of potential of resistance to the problematisation. A key area of resistance would be to shift the focus from addressing the quality of mental health services and clinicians to act “further upstream” through changes to service funding, policy, and social norms to address broader determinants to improve mental health outcomes ([45], p. 844).

### A deficit model of mental health: the choice of outcome measures

Having determined the need for outcome measures in mental health policy and plans, the choice of outcome measures required further problematisation. There are nine measures that are specified for use by Australian public sector mental health services under the National Outcomes and Casemix Collection (NOCC). Table 4 shows the descriptive characteristics of the measures and the settings and Australian States and Territories in which they are used ([13], p. 265). In what follows, we explore the representation of the ‘problem’ of what constitutes a valid measure of mental health service quality using Bacchi’s six questions.

**Table 4:** Descriptive characteristics of mental health outcome measures in Australia

	Outcome measure	State/ territory	Rater	Overarching constructs	No. of core items
Children and adolescents	HoNOSCA	All	Clinician	Range of behavioural, symptomatic, social and impairment domains	15
	CGAS	All	Clinician	Dysfunction	1
	SDQ	All	Consumer and/ or parent	Behaviours, emotions and relationships	25
Adults	HoNOS	All	Clinician	Mental health and social functioning	12
	LSP-16	All	Clinician	Disability	16
	MHI	Qld	Consumer	Psychological distress and well-being	38
	BASIS-32	Vic, Tas, ACT	Consumer	Symptom and problem difficulty	32
	K-10+	NSW, WA, SA, NT	Consumer	Non-specific psychological distress	10
Older adults	HoNOS65+	All	Clinician	Mental health and social functioning	12
	LSP-16	All	Clinician	Disability	16
	MHI	Qld	Consumer	Psychological distress and well-being	38
	BASIS-32	Vic, Tas, ACT	Consumer	Symptom and problem difficulty	32
	K-10+	NSW, WA, SA, NT	Consumer	Non-specific psychological distress	10

NSW, New South Wales; Vic, Victoria; Qld, Queensland; WA, Western Australia; SA, South Australia; Tas, Tasmania; ACT, Australian Capital Territory; NT, Northern Territory

When looking at the choice of outcomes measures, we see a shift from a managerial discourse to a biopsychosocial discourse underpinned by a deficit model of mental health. Analysis of the choice outcome measures in response to Bacchi’s six questions is summarised in Table 5 and discussed below.

**Table 5:** Analysis of the *choice of* outcome measures reported in the documents

Bacchi’s Question	Response
1. What is the problem [of the choice of mental health outcome measures] represented to be?	• The need for mental health services to address individual deficits in functioning
2. What presuppositions or assumptions underpin this representation of the ‘problem’?	Biopsychosocial discourse:
	• Mental ‘illness’ is situated within the individual (biologically, psychologically, and socially), represented by deficits as defined in the chosen outcome measures
	• Quality mental health care addresses deficits in the individual
3. How has this representation of the ‘problem’ come about?	• Historical dominance of the biomedical model of mental illness within mental health disciplines
	• Incorporation of the psychosocial into the biopsychosocial models in the 1970s
	• Supported by neoliberalism as the main approach to mental health care governance
4. What is left unproblematic in this problem representation?	• Silencing of alternative approaches, e.g., strengths-based/recovery-focused services, clinician judgement
	• The role of social determinants of health
5. What effects are produced by this representation of the ‘problem’?	• Stigmatising consumers by constituting people with mental health problems as having deficits that are measured and ‘fixed’ through mental health care
	• Clinician burden to complete the measures, leading to prioritisation of clinician-rated measures and excluding consumer/carer perspectives
	• Lack of focus on and funding for the broader issues affecting mental health
6a. How/where is this representation of the problem produced, disseminated, and defended?	Reports and publications; AMHOCN website and documents; items in the outcome measures; Diagnostic and Statistical Manual of Mental Disorders (DSM)
6b. How can it be questioned, disrupted, replaced?	• Use of strengths-based and recovery-focused measures
	• Measure services rather than consumers
	• Measure social determinants of health

### What is the problem [of the choice of outcome measures] represented to be?

In order to measure the outcomes of mental health services there is a need to determine what constitutes a good outcome in the first place. Here the problem is represented to be the need for mental health services to address individual deficits in functioning. This reflects an “individual-deficit model of diagnosis for mental health [that] asserts that the symptoms of mental illness are the result of personal limitations” ([50], p. 447). Key domains explored in the measures include difficulties or ‘impairments’ in behaviour, psychological functioning, social functioning, and physical health, reflecting a biopsychosocial deficits model of mental health.

The biopsychosocial deficit focus can be seen in wording of the questions included in the measures. For example, the Health of the Nation Outcome Scales (HoNOS) are a suite of measures applicable to different settings (adult, child and adolescent, older adult) where clinicians rate the severity of a range of problems such as: overactive, aggressive, disruptive behaviour; non-accidental self-injury; problem drinking or drug taking; problems with relationships; problems with activities of daily living; etc. The Life Skills Profile (LSP) is another clinician rated measure assessing functioning over the past 3 months and includes questions such as: “Does this person generally have difficulty with initiating and responding to conversation?”; “Does this person generally withdraw from social contact?”; “Does this person generally look after and take his or her own prescribed medication (or attend for prescribed injections on time) without reminding?”

The Basis-32 measure is a consumer-rated measure that similarly focuses on individual problems, asking: “In the past week, how much difficulty having you been having” across 32 areas of life such as managing day-to-day life, work, school, relationships, depression, anxiety, and physical symptoms such as headaches and sleep disturbance, among others. The Kessler-10 (K10) is another consumer-rated measure asking questions about how the person has been feeling. Questions include, for example: “In the last four weeks, about how often did you feel hopeless?”; “In the last four weeks, about how often did you feel worthless?”

### What presuppositions or assumptions underpin this representation of the ‘problem’?

The problem representation for the choice of outcome measures is underpinned by biopsychosocial discourse. Key concepts include ‘problems’, ‘deficits’, ‘difficulties’, ‘biomedical’, ‘psychological’, and ‘social’, which create categories of mental health problems (e.g., depression, anxiety, etc.), the degree of problems or deficits related to these diagnoses, and aspects of the person’s life in which these problems occur (within their biology, their psychology, and/or their relationships). From this come the binaries of being a person with or without a mental health diagnosis and experiencing or demonstrating normal versus abnormal cognitions, behaviours, and psychosocial functioning.

This representation of the problem is underpinned by two main assumptions. The first is that mental ‘illness’ is situated within the individual (biologically, psychologically, and socially), represented by a specific range of deficits as defined in the chosen outcome measures. Some measures incorporate social determinants such as living conditions and occupation. For example, the HoNOS includes the questions ‘Problems with living conditions and daily domestic routines’ and ‘Problems with occupation, activities in daytime environment’. However, the focus remains on the individual rather than the broader socio-political or structural aspects of social determinants. The second assumption is that the role of quality mental health care is to address deficits in the individual (in relation to their biological, psychological, and social functioning) as opposed to, for example, examining and addressing issues in health services or the broader socio-political context of mental health.

### How has this representation of the problem come about?

There is a long history of focusing on individual deficits within mental health disciplines (psychiatry, psychology, nursing, etc.) underpinned by the dominance of the biomedical model of mental illness. The biomedical model sees mental health as akin to physical health in defining and understanding illness. As with the biomedical model of physical health, the aim is to explore signs and symptoms of illness in the individual and attempt to treat these. The extension of the biomedical model to incorporate the psychosocial (the biopsychosocial model) in the 1970s extends the identification of signs and symptoms to the person’s psychology and broader social factors yet continues to locate the cause of problems within the individual. The focus on biopsychosocial deficits in the individual is furthermore supported by the predominance of neoliberalism in mental health care. According to Esposito & Perez ([51], p. 414):

… this obsession with medicalization and the tendency to treat ‘mental illness’ as a problem within the individual continues to be supported within the prevailing neoliberal logic that downplays the social realm, treats individuals as self-contained agents, and pathologizes thoughts and behaviors that deviate from what the market defines as functional, productive, or desirable.

The dominance of the biopsychosocial, deficit model of mental illness in the choice of outcome measures can be seen in the initial process of determining which measures would be included. The first report on the measurement of consumer outcome in mental health [32] outlines the criteria for inclusion in the suite of national measures. The report states: “the measure of outcomes should be multidimensional, covering symptoms and disability” (p. 29). Disability is outlined in terms of “the symptoms of the individual consumer [and] … the consequences of the illness especially with regard to the ability of the consumer to function in the community and to maintain independence from the mental health services” (p. 29), reflecting a neoliberal focus on productivity and individual responsibility for health and wellbeing [40].

Further criteria were that measures should be applicable, acceptable, practical, reliable, valid, and sensitive to change [32]. Early consultation identified the importance to clinicians, consumers, and other stakeholders that symptoms, disability, and consumer satisfaction were important elements of mental health outcomes. Measures that are ‘applicable’ are those that address these dimensions. Acceptability referred to measures being brief and user-friendly. Practicality included minimal cost, ease of scoring and interpretation, and minimal need for training. Reliability and validity relate to the measures having acceptable psychometric properties. Finally, sensitivity to change means that a change in the measure can be interpreted in relation to change in an external criterion. These criteria were then used to search the literature on existing measures to create a shortlist of potential measures [52]. This process ultimately limited the way in which outcomes could be defined and measured to a biopsychosocial, deficit-based definition of outcomes measured within a managerial focus on time and cost-effectiveness.

### What is left unproblematic in this problem representation?

The current representation of the ‘problem’ of what constitutes a valid measure of mental health service quality focuses on the degree to which deficits within individual functioning are addressed through treatment. This problematisation silences alternative problem representations. For example, aspects of mental health that do not meet the criteria outlined above are silenced in the current problematisation. This includes strengths-based and recovery-focused outcome measures [13, 47, 52], as well as the validity of qualitative measures such as clinician judgement or outcomes relating to consumers’ personal experiences of meaningful changes. Measurement of the functioning of the services themselves, such as the role of service culture, staffing, or the extent to which services are recovery oriented [53], while included to some extent by AMHOCN as part of the mental health information development strategy (e.g., using the Your Experience of Service Survey and the Carer Experience Survey), are not part of the NOCC and therefore also largely silenced.

The problem representation furthermore leaves the role of social determinants of mental health unproblematic. For example, questions relating to occupation or living conditions focus on the availability of work opportunities, adequate, safe housing, or access to other resources such as transport at the individual level rather policies, social norms, and structural causes. The potential of measuring and addressing the social determinants affecting individuals and their mental health more broadly is therefore silenced in the current focus on individual deficits in biopsychosocial functioning [51, 54].

### What effects are produced by this representation of the problem?

The current representation of the problem has a range of effects on consumers, carers, clinicians, and services. A significant effect of the current problematisation is that it constitutes people with mental health problems as having deficits and as passive recipients of mental health care through which these deficits are measured and ‘fixed’. A deficit approach has lived effects by stigmatising consumers and sustaining “public practice which divide between ‘us’ and ‘them’” ([55], p. 1).

The number of measures required for ROM furthermore places burden on time-poor clinicians. This has led to a tendency of clinicians to prioritise completing clinician-rated measures, such as the HoNOS measures, which are “administered at a much greater rate than consumer measures” ([13], p. 267). This has the effect of excluding consumer perspectives regarding mental health outcomes.

It is widely recognised that action needs to be taken at the individual, health services, and societal levels if we are to achieve the policy aim of reducing the prevalence and severity of mental health problems [56]. The focus on the individual and their ‘deficits’ prioritises ‘fixing’ individuals and their relationships rather than the broader issues that affect mental health. Adversity is framed as “an individual matter which excludes the possibility that adversity could result from circumstances which require social rather than individual change” ([40], p. 13). The effect of this is that funding and action is focused on the individual and health services levels rather than on the broader forces affecting mental health.

### How/where is this representation of the ‘problem’ produced, disseminated and defended and how could it be questioned, disrupted and replaced?

The representation of the problem of what to measure in determining mental health outcomes is produced and legitimised in the reports and publications relating to the introduction of ROM in mental health care in Australia (e.g., [5, 13, 32, 33]). It is also represented on the AMHOCN website and in the documents provided on the website (e.g., in documents providing an overview of the measures and in the training manuals for the use of each of the measures), as well as in the measures themselves and the literature on the production and validation of the measures. Underpinning these is the focus on individual biopsychosocial deficits in the Diagnostic and Statistical Manual of Mental Disorders (DSM). The DSM is a primary authoritative text used to define mental disorders and which “singularly focus[es] on individuals and exclude[s] social and structural determinants of mental health” ([57], p. 646).

The biopsychosocial representation of the ‘problem’ of mental health problems can be disrupted through a shift in focus to strengths within individuals and their relationships. For example, a review by the National Mental Health Information Development Expert Advisory Panel identified concern with the lack of strengths-based language in Australia’s mental health outcome measures and advised a shift in focus to measuring functioning in terms of “personal recovery, social recovery and clinical recovery” ([52], p. 339). An example of an alternative way to measure mental health outcomes is the Warwick-Edinburgh Mental Well-being Scale (WEMWBS), which was developed in Scotland to address the issue with deficit focused measures [53]. The WEMWBS uses positively worded statements such as “I’ve been feeling optimistic about the future”, “I’ve been thinking clearly”, etc. Measures of recovery, such as those measuring dimensions of CHIME (Connectedness, Hope and optimism about future, Identity, Meaning of life, and Empowerment [58]); or the use RAS-DS (Recovery Assessment Scale-Domains and Stages [59]; measuring the domains of ‘Doing things I value’, ‘Looking forward’, ‘Mastering my illness’, and ‘Connecting and belonging’), could also be used to measure outcomes of mental health services. However, while a focus on strengths and recovery could disrupt the deficit focus of current measures, this approach continues to focus on individual functioning at the expense of the role of social determinants of mental health [57].

Measuring the functioning of the services is a further opportunity for disrupting the focus on individual functioning. The Scottish Recovery Indicator (SRI), used in assessing mental health care in Scotland, is an example of this approach. The SRI is used by services as a self- assessment tool “measuring the extent to which services are implementing a recovery-oriented practice model within their work” ([53], p. 23). In the UK, INSPIRE, https://www.researchintorecovery.com/measures/inspire/, is another validated tool that assesses recovery support from workers. A further disruption is through a focus on measuring the social determinants of mental health, such as measures of income inequality, financial inclusion, equity, etc. [53]. Finally, ensuring services provide interventions that are rights based and address social determinants is another potential disruptor. Such examples would include Individual Placement and Support, an evidence-based employment program that supports people living with a serious mental illness to find and maintain work [60], and Housing First, an evidence-based housing model [61].

## Discussion

Bacchi’s [23] analytic framework was used to facilitate an in-depth understanding and critique of the introduction of ROM to evaluate Australian public mental health services. This analysis identified the managerial discourse of mental health service quality and accountability, and the dominance of deficits-based models in mental health care, as underpinning and informing the use and selection of outcome measures. As discussed, implications of these problematisations are significant for all stakeholders in the context of mental health care provision, and particularly those individuals seeking care and treatment from services. This is not to suggest that efforts to improve the quality of mental health services, including through the use of ROM, should be abandoned. Rather, the purpose is to open up the possibility of thinking different about the problem to support future efforts to improve care and support.

The rise of managerialism in health and social care supported by neoliberal modes of governance has attracted a great deal of discussion [62]. Our analysis of the problem representation of the need for ROM adds to this literature by highlighting the assumptions underpinning the decision to use ROMs in mental health, the potential consequences, and the exclusion of other problematisations, such as the role of social determinants of health and mental health service funding. The decision to routinely measure mental health outcomes and use these in benchmarking and funding allocation aims to improve mental health services and consumer experiences of care. However, the potential (unintended) consequences of the problematisation underpinning their use are important to consider in future efforts to ensure mental health services are in fact meeting the needs of the communities they service. A recent policy analysis that examined gaps and barriers to addressing social determinants of health (SDoH) for people living with a disability in Australia found that a “services drift” was occurring in policy [63]. This drift was resulting in a focus on “improv[ing] experience of and access to health services … even when there is an acknowledgement that broader SDoH are not being adequately addressed” (p.226). Similar to our analysis, Green at al. [63] attribute this drift to human biases, the biomedical model, and neoliberal discourses of individualism.

Our analysis furthermore highlights the need to reconsider the focus of individual deficits when measuring outcomes. Internationally there is broad acknowledgement of the need for a paradigm shift within mental health care service provision. The World Health Organization (WHO) identifies respect for legal capacity, non-coercive practices, participation, community inclusion, and a recovery approach as critical areas for mental health services [64]. The Special Rapporteur to the Human Rights Council has advocated that “distress, treatment and support must be seen more broadly and move far beyond a biomedical understanding of mental health” ([65], p.1).

In the Australian context, the recent Royal Commission into Victoria’s Mental Health System [66] is influencing service reform. For example, the Office of the Chief Psychiatrist (OCP) [67] announced that the South Australian Law Reform Institute was asked to ‘Consider the findings of the Royal Commission into Victoria’s Mental Health System’ in their current review of the Mental Health Act 2009. Additionally, the OCP [68] referenced the need to consider the Royal Commission recommendations in the development of the Rehabilitation Model of Care, which relates to 72 newly funded rehabilitation beds. Recommendations from the Victorian Commission include emphasising personal recovery over clinical recovery and acknowledging the impact of people’s living and working conditions on their health and well-being, and specifically, addressing housing needs. We advocate that within this climate of reform, the use and selection of mental health outcome measures is properly reviewed and addressed to better align to services that are rights based, person-centred, and recovery focussed, taking into account the effect of broader social determinants on mental health and mental illness.

There is a high degree of interest in routine outcome monitoring within mental health services internationally [69–71]. Roe at al. [3] highlight that mental health systems’ endeavours to become outcome orientated is a process rather than an endpoint. We would agree with this sentiment. The significant changes in understandings of mental illness and distress (e.g., social determinants versus biogenetic causation) and approaches and interventions (e.g., the growing evidence base for lived experience workforce and services) requires ongoing review and updating of outcome measures that are used to inform and measure care.

Obvious gaps in the Australian public sector context are measures of recovery and consumer experiences of services. A recent systematic review of patient reported outcomes measures and clinician assessments in mental health care found that the most represented categories included psychosocial functioning and impairment and symptom severity and distress [2]. Consumer needs were rated by 17% (of services) by clinician-rated measures, and 9% by consumer-rated measures. This is concerning given the reported discrepancy often observed between consumer versus clinician rated measures [71], and the expectation that people are active participants in their own care. Measures of consumer satisfaction with services (Patient Reported Experiences of Services- PREMs) and recovery were significantly less represented [2], further highlighting the misalignment of outcome measures with contemporary care models that are recovery-orientated and trauma informed. PREMs are critical to informing service reviews and improvements, and actively facilitate inclusion of lived experience [69]. Collecting information on what is important to consumers should positively inform care and ongoing service development [71]. While PREMs are currently collected through the Your Experience of Service (YES) Survey and the Carer Experience Survey (CES), they are not part of the NOCC.

National mental health policy effort and support and mental health service structures are key factors in the implementation of ROMs [3]. In the Australian context, AMHOCN have led reform related to the use and selection of mental health outcome measures including the development of a new measure, the Living in the Community Questionnaire (LCQ), that measures social inclusion [72]. However, implementation is complex [73] and remains dependant on national and State and Territory level support and leadership. Future work should also include strategies and processes to meaningfully engage services in using data to drive practice change, such as through ongoing cycles of implementing and evaluating change, and provision of funding to support practice improvement. Smith-Merry and colleagues [53], for example, discuss the importance of a ‘communicative approach’ to the use of ROM in mental health in Scotland, contrasting this to the “culture of targets and terror for coordinating the work of the NHS” (p. 2) in England.

In the current climate there is an acknowledgement that broader government support is required to ensure people living with mental illness and distress receive the necessary resources and supports to facilitate recovery [66, 74]. Cross sector policies and partnerships are required to address socioeconomic disadvantage and the associated broad range of needs, including physical health, housing, education, and employment [74]. This complexity and need for broader government response needs to be captured in the dialogue regarding the use of mental health outcome measures that currently lays the responsibility for change at the service and individual level of consumers and clinicians.

Alternatives to the current representation of the ‘problem’ of mental health outcome measures in Australia, such as measuring and addressing broader determinants of mental health and a focus on consumer experiences, are important considerations in the future of ROMs in mental health care. However, we must not forget to follow Bacchi’s advice to apply the six questions of the ‘What is the problem represented to be’ approach to our own problem representations. This will help to ensure that “we do not simply buy into [alternative] problem representations without reflecting on their origins, purposes and effects” ([23], p.19).

### Limitations

This study is a critical analysis of Australian policy relating to the introduction and use of routine outcome measures in public mental health services. As such it is limited in its focus on the Australian context, and specifically, the public health sector. The authors acknowledge that in the Australian context non-government organisations have a significant role in supporting people living with a mental illness, and that these organisations may select a different array of outcome measures (e.g., the Camberwell Assessment of Needs). This disparity in use of measures across sectors potentially introduces further complexity to the issue. Although exploration of this is not within the scope of this paper, we acknowledge that this is an important factor to consider in any potential review and/or reform of public mental health services’ ROMs.

## Conclusion

Through the use of Bacchi’s [23] ‘What is the problem represented to be’ approach to analyse Australia’s policy of ROM in mental health services, it is evident that managerial and biopsychosocial discourse underpin policy in this area. While the purpose of ROM is to improve mental health care and consumer outcomes, this analysis highlights the (unintended) consequences of the decision to adopt ROM and the specific measures chosen. Framing the problem of mental health outcomes within managerial and biopsychosocial discourses locates the problem within individual health providers, services, and consumers. Lack of positive mental health outcomes are therefore seen to result from deficits in health professionals/ services and in individual consumers and their relationships. The use and choice of ROMs in Australian mental health services therefore shifts the focus away from broader determinants of mental health and mental health care, such as service funding, recovery-oriented care, and the social determinants of mental health. The use of ROM in mental health is increasing worldwide. Adopting a critical lens to explore Australia’s policy of ROM in public mental health services opens up the possibility of thinking differently about outcome measurement in the mental health context.

## Data Availability

The datasets used and/or analysed during the current study are available from the corresponding author on reasonable request.

## Abbreviations

AMHOCN: Australian Mental Health Outcomes and Classifications Network
BASIS-32: Behavior & Symptom Identification Scale 32
CES: Carer Experience Survey
CGAS: Children’s Global Assessment Scale
HoNOS: Health of the Nation Outcome Scale
K-10+: Kessler − 10+
LSP-16: Life Skills Profile 16
MHI: Mental Health Inventory 38
ROM: Routine Outcome Measures
SDQ: Strengths and Difficulties Questionnaire
YES: Your Experience of Service Survey
